# Antidepressant-Like Effects of Coumaroylspermidine Extract From Safflower Injection Residues

**DOI:** 10.3389/fphar.2020.00713

**Published:** 2020-06-17

**Authors:** Shifei Li, Ting Li, Yufang Jin, Xuemei Qin, Junsheng Tian, Liwei Zhang

**Affiliations:** ^1^Key Laboratory of Chemical Biology and Molecular Engineering of Education Ministry, Institute of Molecular Science, Shanxi University, Taiyuan, China; ^2^Modern Research Center for Traditional Chinese Medicine, Shanxi University, Taiyuan, China

**Keywords:** coumaroylspermidine extract (CSE), safflower injection, residues, anti-depressant, chronic unpredictable mild stress

## Abstract

In this study, a total coumaroylspermidine extract (CSE), which included four coumaroylspermidine compounds, was prepared from safflower injection (a traditional Chinese medicine) residues for the first time. The total content of the four coumaroylspermidine compounds was determined to be 64.86 ± 0.41% using high-performance liquid chromatography. We then evaluated the anti-depressant effect of CSE by using a chronic unpredictable mild stress (CUMS) model in rats. Results of sucrose preference tests, open field tests, and forced swimming tests suggest that CSE exhibits a significant anti-depressant effect. In studies to explore the mechanism, CSE was found to inhibit the increases in levels of corticosterone and decreases in levels of 5-hydroxytryptamine, dopamine, and noradrenaline induced by CUMS. Metabolic profiling showed that 10 endogenous metabolites and four metabolic pathways were altered after CSE treatment. Thus, this study not only found a spermidine extract with antidepressant effect from safflower injection residue for the first time but also provided a way for the efficient utilize of safflower injection residue.

**Graphical Abstract:**
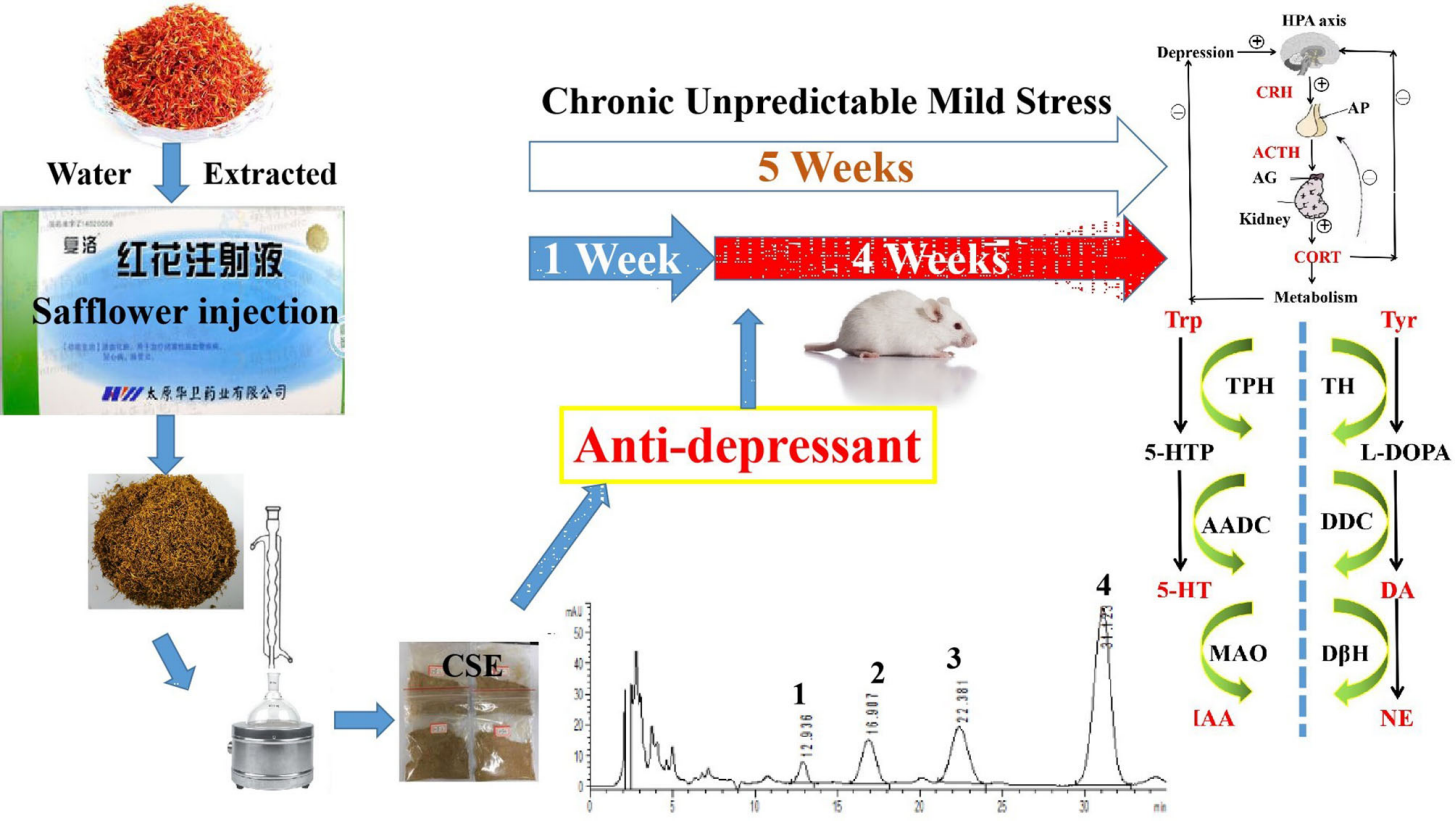
Antidepressant-like effects of the coumaroylspermidine extract from a traditional Chinese medicine (safflower injection) residues.

## Introduction

The influence of traditional Chinese medicine (TCM) is increasing throughout the world along with the concern that the TCM residues (TCMRs) may undermine our environment (Ma et al., 2019). Over the past few decades, the volume of TCMR disposal has soared, exceeding 70 million tons per year in China on average (Wang and Feng, 2009; Zhou et al., 2018). It has been reported that after water or ethanol extraction, traces of active components may still remain in the waste TCMR (Liu et al., 2016; Wang et al., 2018). However, using current methods, TCMRs are generally disposed of after the target ingredient has been extracted since the residues are regarded as byproducts of TCM. For example, some TCMRs are used as bulking agents for food waste composting, while active ingredients, such as proteins, still remain in the residues (Ma et al., 2019). The yield of polysaccharides extracted from *Schisandra chinensis* residues could reach ~5% using a suitable extraction method (Youn and Noh, 2001; Zhou et al., 2014). Furthermore, TCMRs which contain high levels of water along with some nutrients are routinely discarded with solid wastes, leading to serious environmental pollution (Tian et al., 2017; Zhang et al., 2017). Therefore, the rational recycling of TCMRs and the scaling-up of this practice within the industry are important to the sustainable development of the environment.

The Chinese Ministry of Health has recorded Safflower injection as an official drug, and it is used to treat occlusive cerebrovascular disease, coronary heart disease, and vasculitis. It is prepared with the processes of water extraction and alcohol precipitation in which hydroxyl safflower yellow-A and total flavonoids are used as quality control standards (Ao et al., 2018). This process generates residues containing high concentrations of the active compounds. It would therefore be beneficial to study the drug residue in order that it can be utilized rather than wasted.

Spermidine is a naturally occurring polyamine that is involved in diseases, including nerve injury, cardiovascular and muscle-related disease, autophagy and mitophagy induction, and inflammation (Eisenberg et al., 2009; Madeo et al., 2018). In 2018, a review titled “Spermidine in health and disease” was published in the journal *Science*. In this review, authors discuss the properties of spermidine as a well-tolerated caloric restriction mimetic and its uses for targeting various age-associated adversities from molecular and physiological perspective (Madeo et al., 2018). However, whether spermidine is effective in depression has not been reported in the literature.

**Figure 1:**
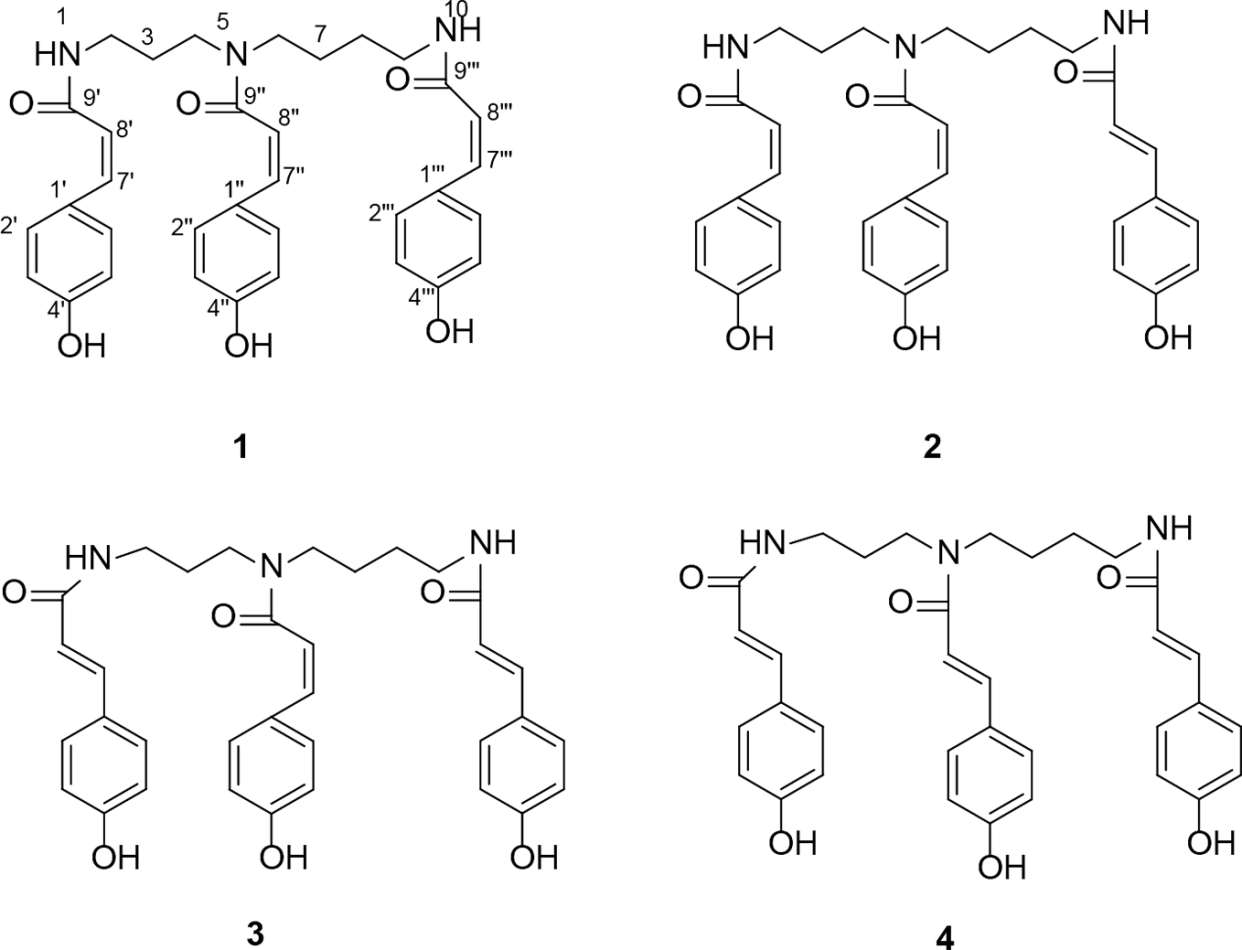
The structure of four coumaroylspermidines in CSE. N1,N5,N10-(*Z*)-tri-p-coumaroylspermidine (**1**), N1,N5-(*Z*)-N10-(*E*)-tri-p-coumaroylspermidine (**2**), N1-(*E*)-N5-(*Z*)-N10-(*E*)-tri-p-coumaroylspermidine (**3**), and N1,N5,N10-(*E*)-tri-p-coumaroylspermidine (**4**).

In our previous study, we obtained four coumaroylspermidine compounds with three different coumaroyl groups (**1**: N1,N5,N10-(*Z*)-tri-p-coumaroylspermidine, **2**: N1,N5-(*Z*)-N10-(*E*)-tri-p-coumaroylspermidine, **3**: N1-(*E*)-N5-(*Z*)-N10-(*E*)-tri-p-coumaroylspermidine, and **4**: N1,N5,N10-(*E*)-tri-p-coumaroylspermidine) from safflower injection residues (structure see **Figure 1**). These compounds showed clear inhibition of 5-HT reuptake in rat brain synaptosomes, demonstrating that proper recycling of residues can yield active components that could have therapeutic value (Yuan et al., 2015). In this study, we obtained a total coumaroylspermidine extract (CSE), which included four coumaroylspermidine compounds from safflower injection residues. The total coumaroylspermidine content of CSE was 64.86 ± 0.41%, and the yield of CSE exceeded 85%. In addition, CSE has shown antidepressant effects in the pre-experiment on tail suspension test in mice. It is known that depression is a chronic psychological disease with a genetic tendency caused by a combination of factors. The pathogenesis and pathophysiological characteristics of depression are still not very clear, but disturbances of neurotransmitters are considered to be the main pathogenesis of depression (Palazidou, 2012; Joca et al., 2015). Therefore, we speculated that CSE may have antidepressant effects, and we used the CUMS model to evaluate its antidepressant effect and study its mechanism of action. It is hoped that this study will not only discover the depressive effect of CSE but also provide a way for the high added value utilization of safflower injection residues.

## Materials and Methods

### Chemicals and Materials

Column chromatography (CC) was performed on RP-C18 silica (50-75 µm, Merck). Venlafaxine hydrochloride capsules were purchased from Chengdu KangHong Pharmaceutical Group Co., Ltd. (No. 180501). D_2_O (Merck reagents Co., Ltd., America), sodium carboxymethyl cellulose (CMC-Na, Tianjin Kaitong Chemical Reagent Co., Ltd, China), chloral hydrate (analytical grade), chromatography-grade methanol, high-performance liquid chromatography (HPLC)-grade acetonitrile, and HPLC-grade methanoic acid. ELISA kits for corticosterone (CORT), adrenocorticotropic hormone (ACTH), and corticotropin-releasing hormone (CRH) were acquired from Westang Biological Technology Co., Ltd (Shanghai, China). Safflower injection residues were acquired from Shanxi Huawei Pharmaceutical Co., Ltd, which is the main manufacturer of safflower injection (batch number SHHW20161108065T). Safflowers were collected from Yumin County, Xinjiang Province (Geographical coordinates are 82°15'00''~83°30'00''East longitude, 45°24'00''~46°30'00''North latitude). Standards of the compounds (**1**): N1,N5,N10-(*Z*)-tri-p-coumaroylspermidine, (**2**): N1,N5-(*Z*)-N10-(*E*)-tri-p-coumaroylspermidine, (**3**): N1-(*E*)-N5-(*Z*)-N10-(*E*)-tri-p-coumaroylspermidine, and (**4**): N1,N5,N10-(*E*)-tri-p-coumaroylspermidine were prepared by our group to a purity of >95%.

### Preparation of CSE

Dried safflower residues (200 g) was refluxed with 75% EtOH (6 L × 3 × 2 h). The extraction is filtered and then concentrated to 200 mL with a rotary evaporator at 50 °C and centrifuged to obtain the precipitate. The precipitate was dissolved in 20% ethanol and then subjected to RP-C18 column chromatography eluted with H_2_O and a step gradient of EtOH to produce five fractions: Fr1 (pure H_2_O), Fr2 (H_2_O−EtOH=8:2), Fr3 (H_2_O−EtOH=1:1), Fr4 (H_2_O−EtOH=2:8), and Fr5 (pure EtOH). F3 was concentrated and dried to obtain the CSE (yellow powder). The total content of the coumaroylspermidine (including compounds **1**–**4**) in the CSE was determined by HPLC. The total yield of coumaroylspermidine compounds was calculated with the following equation:

$$\text{Yield }\left(\%\right)\text{ = }\frac{\Sigma \text{ total content of the coumaroylspermidine for CSE}}{\sum \text{total content of the coumaroylspermidine for safflower injection residues}}*100\%$$

### Determination of Total Coumaroylspermidine Compound Content

A method including sample preparation and method validation was previously determined by our group (Li et al., 2016). The total coumaroylspermidine compound content of CSE was measured by a HPLC-DAD machine (Agilent 1260). The column was carried out on a Zorbax XDB C18 column (250 × 4.6 mm, 5 μm). The mobile phase was methanol-water with a volume ratio of 55% (MeOH:H_2_O), and the detection wavelength was 270–300 nm. The column temperature was 25 °C. Flow rate and injection volume are 1 mL/min and 10 ml, respectively. The calibration curves of the four coumaroylspermidine compounds are provided in the supporting material.

### Animals

A total of 60 adult male SD rats (weighing 180–220 g) were purchased from Beijing Vital Laboratory (license number SCKX-2016-0006) and habitualized to a new environment for 1 week. Five rats were placed in each cage under controlled breeding room conditions (temperature: 24 ± 1°C, humidity: 60 ± 5%) with free access to food and water. The animal study was reviewed and approved by the Experimental Animal Ethical Committee of Shanxi University. All experimental procedures in the present study were performed in accordance with the NIH Guide for the Care and Use of Laboratory Animals (United States) and the Prevention of Cruelty to Animals Act (1986) of China.

### Chronic Unpredictable Mild Stress (CUMS) Procedures

**Figure 2:**
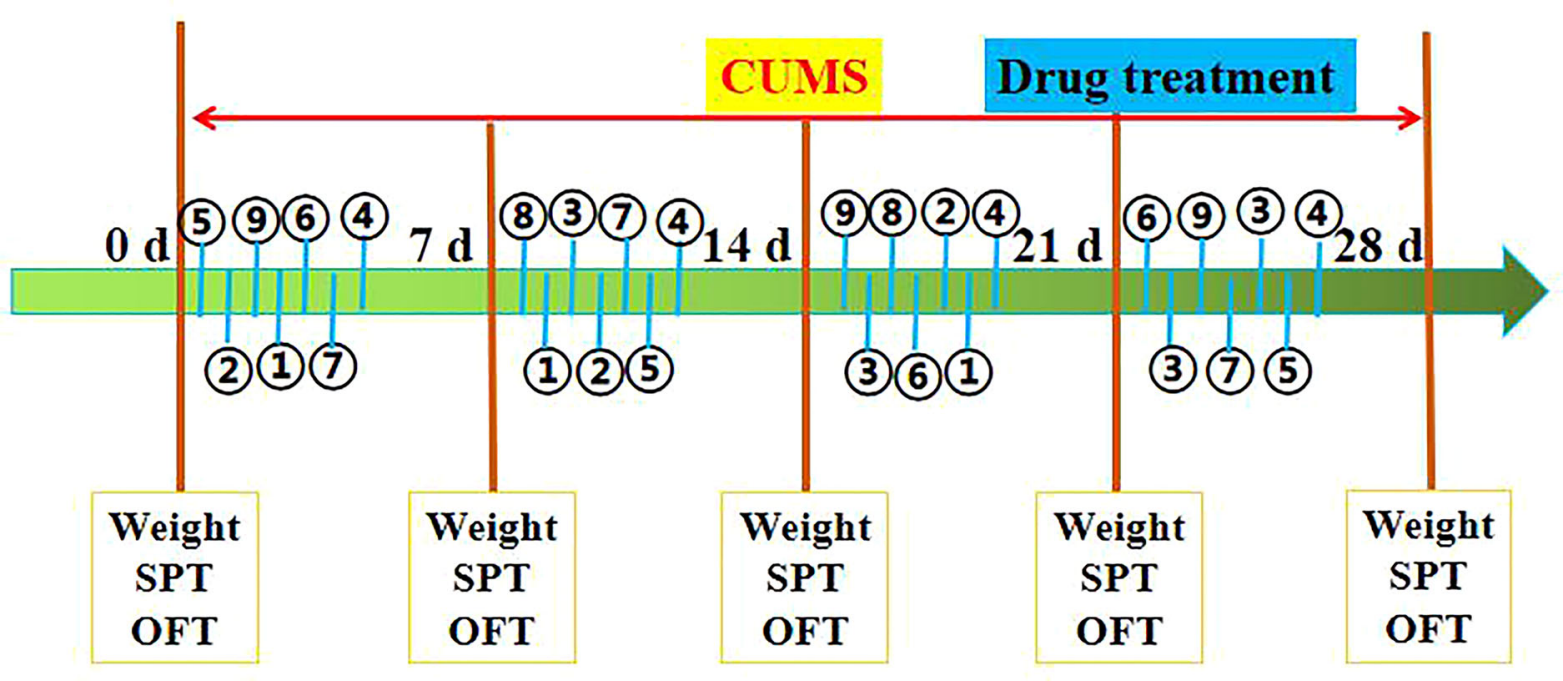
Schematic diagram of the experimental procedure, as described in *Materials and Methods*. Stimulating factors, including ① tail pinch, ② cold swimming, ③ food deprivation, ④ water deprivation, ⑤ exposure to a hot environment, ⑥ day and night reversal, ⑦ foot shock, ⑧ binding of bottles, and ⑨ ultrasonic stimulation. SPT (sucrose preference), OPT (open field test), and Weight (body weight). The rats were sacrificed on 29th day.

CUMS procedures were carried out as described previously with some modifications (Tian et al., 2015). The rats were exposed to the CUMS model for 4 consecutive weeks throughout the experiment. The CUMS procedures consisted of nine stressors: (1) 2 min tail pinch (1 cm from the proximal end of the tail), (2) 5 min cold swimming at 4°C, (3) 24 h of food deprivation, (4) 24 h of water deprivation, (5) 5 min of exposure to a hot environment (45°C), (6) 24 h of day and night reversal, (7) a foot shock (36 mV with a duration of 10 s for each shock), (8) binding of bottles (3 h), and (9) ultrasonic stimulation (3 h). All rats in the CUMS groups were under the same combination of stressors at the meantime. All rats received each a random combination of stressors 1–9 in a week (Detailed information about the use of stimulating factors in the experiment is shown in **Figure 2**).

### Drug Treatments

Sixty rats were randomly divided into six groups, 10 rats in each group: (1) control group (Con): without any treatment or stress; (2) CUMS model group (Mod): only CUMS stress; (3) low dosage of CSE (8.65 mg/kg body weight, CSE-L) group: CSE-L treatment and CUMS stress; (4) middle dosage of CSE (17.30 mg/kg body weight, CSE-M) group: CSE-M treatment and CUMS stress; (5) high dosage of CSE (34.60 mg/kg body weight, CSE-H) group: CSE-H treatment and CUMS stress; and the (6) venlafaxine group (35.00 mg/kg body weight, Ven): venlafaxine treatment and CUMS stress. CSE was made suspension with CMC-Na and distilled water, which was given by intragastric administration every morning at 7:30–8:00 and continued for consecutive 4 weeks. Both Con group and Mod group were given the same volume of saline. All ten rats in each group were subjected to all behavioral tests. After the experiments, the brains and serum were collected for following examination.

### Behavioral Tests

#### Body Weight

The body weight measurements were respectively taken on week 0 (before CUMS procedure) and weeks 1, 2, 3, and 4 (during and after the CUMS procedure).

#### Sucrose Preference Test (SPT)

There was seventy-two hours before the test. During the acclimation period, within the first 24 hours, rats were free to obtain two bottles of 1% sucrose solution, and within the second 24 hours, one cup of sucrose solution with tap water was provided instead. Then for the third 24 h, the rats were deprived of food and water. The test procedure was the same as the previous study (Tian et al., 2013), whereby rats were given a bottle of tap water and a bottle of sucrose solution respectively for baseline measurement of sucrose preference. After 4 h, the sucrose preference rate was calculated using the following equation:

$$\text{sucrose preference rate = }\frac{\text{sucrose intake }}{\text{total liquid intake}}*100\%$$

The SPT was conducted respectively on weeks 0, 1, 2, 3, and 4 (before, during, and after CUMS procedure).

#### Open Field Test (OFT)

Locomotor activity was assessed using the open field test as described previously (Tian et al., 2013). The instrument consisted of a black iron box (100 × 100 × 40 cm), with the box bottom divided into 5*5 = 25 parts of equivalent size marked with white lines, and the middle one is the central square. Each rat was positioned at the center and allowed free exploration for 5 min. The number of crossing, the number of rearings, and the off-grid time of the rats were tested and recorded in next 4 minutes. The number of crossing was the number of rats crossing grids (means animals' ability to move autonomously). The number of rearings was number of times about rat's paws lifted away from ground (means rats' curiosity of fresh environments). The off-grid time was time from putting the rat into the central grid to escape from the central grid (means animals' ability to adapt to the novel environments). An infrared camera and video system were applied to detect the indicators three times, and the data was analyzed by a behavior analysis system (SLY-ETS, Beijing Shuo Lin Yuan Technology). The OFT was conducted on weeks 0, 1, 2, 3, and 4.

#### Forced Swimming Test (FST)

We referred to the FST in the previous literature and modified it slightly (Gong et al., 2019). We placed the rat in a glass bottle with a height and diameter of 100 cm and 20 cm, respectively. Water at 25°C with a height of 35 cm was placed in the glass bottle. The entire FST experiment lasted 5 minutes, and then the immobility time of the last 4 minutes was recorded using FST software. The immobility time was tested when the rat heads floated above the water during the last 4 min and had ceased struggling. This test was conducted in week 4.

### Detection of Serum CRH, ACTH, and CORT Levels

The serum samples (stored at -80°C) were centrifuged at 5,000 rpm for 15 min at 4°C. The levels of corticotropin-releasing Hormone (CRH), adrenergic Hormone (ACTH), and corticosterone (CORT) in the serum were tested by commercial Enzyme-linked immunosorbent (ELISA) kits (Westang Bio-tech Co, Ltd., Shanghai, China).

### Detection of Monoamine Neurotransmitters Levels

The monoamine neurotransmitters levels in the hippocampus were detected by a LC-MS/MS-IT-TOF. The method was as follows: a 30 mg sample of frozen hippocampus tissue was removed and placed into a glass homogenizer. A pre-chilled 0.1 mol·L^-1^ perchloric acid solution containing 0.01% EDTA-2Na was added. The homogenate was placed into 1.5 mL EP tubes and centrifuged at 15,000 r·min^-1^ at 4°C for 20 minutes, and the supernatant removed. After filtering the supernatant with a 0.22 μm filter, monoamine neurotransmitters including 5-hydroxytryptamine (5-HT), 5-hydroxyindoleacetic acid (5-HIAA), dopamine (DA), glutamic acid (Glu), tryptophan (Trp), tyrosine (Tyr), 3,4-dihydroxybenzylamine hydrobromic acid (GABA), and noradrenaline (NE) were detected in the brain tissue on the LS-MS/MS-IT-TOF machine.

An Agilent ZORBAX SB-C18 column (T3, 2.1 × 100 mm × 1.8 μm) was used in this experiment. The mass spectrometer used an electrospray ionization source (ESI), simultaneous scanning of positive and negative ions, and multiple reaction monitoring (MRM) mode; the source injection voltage was 5,500V (-4,500V), the heating temperature was 550°C, and it had the appropriate pressure of 160 psi of gas. The co-solvent was polysorbate-80 (1psi = 6.895 kPa), and the gas was 265 psi.

### Serum Sample Preparation and ^1^H-NMR Spectrometry

The serum was taken out of storage at -80°C and defrosted in an ice-water mixture left at room temperature. Then, 450 µL serum was mixed with 350 µL D_2_O in a 1.5 mL EP tube and centrifuged for 20 min at 13,000 rpm and 4°C. Following this, 600 µL of supernatant was aspirated and placed in a 5 mm-NMR tube.

^1^H-NMR spectrometry of the serum was measured with a Bruker 600-MHz Spectrometer (Bruker, Germany) at 25°C. The other conditions were as follows: adapting CPMG (Carr-Purcell-Meiboom-Gill) pulse sequence, detection spectrum of 7149.2000 Hz, scanning number of 64.0000, FID resolution of 0.1880 Hz, pulse time of 9.9500 s, and sampling point of 32768.0000. All spectra were subjected to Fourier transforms of ^1^H-NMR spectra in serum using MestReNova NMR data processing software. The spectra were corrected for chemical shifts with creatinine (δ 3.04 ppm) as the standard, and the interval of δ 4.30 ~ 5.16 ppm was the cutoff for the water peaks. The NMR spectrum of δ 0.70 ~ 8.60 ppm was divided into equal widths and segmented and integrated in units of 0.01 before which the data was normalized. The processed data were saved in an Excel sheet for multivariate statistical analysis.

After using SIMCA-P14.1 to centralize and normalize the integral data, the partial least squares method (PLS-DA) was used for group comparison and model verification; the orthogonal partial least squares method was used for discriminant analysis (OPLS-DA) methods. Differential metabolites between groups were compared. SPSS 21.0 and GraphPad Prism 5 were used for statistical analysis and graphing of potential biomarkers. One-way analysis of variance (ANOVA) and t-tests were used to compare differences.

### Statistical Analysis

Values from behavioral data are showed as mean ± SEM. The *t*-test was used to analyze the statistical differences between two groups, and the statistical difference analysis between multiple groups used one-way ANOVA. The threshold for statistical significant was considered to be *P* < 0.05 (*P* < 0.01). Statistical analysis was performed by a SPSS 21.0 software. Pearson's correlation analysis was used to valuate correlations between monoamine neurotransmitters, HPA axis hormones, and differential metabolites.

## Results

### Preparation and Determination of CSE

**Figure 3:**
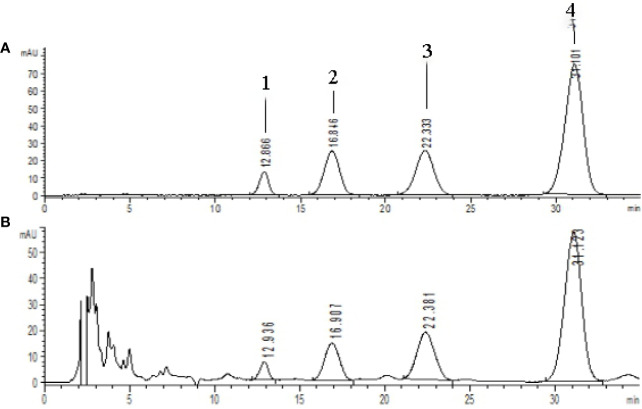
HPLC chromatograms of standards and CSE [λ=280 nm, **(A)** coumaroylspermidine standards, **(B)** CSE]. Compouds **1**-**4** represents the corresponding four coumaroylspermidine products.

The extraction and enrichment process of CSE from safflower injection residues have been detailed in our previous work (Jin et al., 2018). In this study, CSE was prepared three times through the same process. The total content of the four coumaroylspermidine compounds in the CSE, (**1**): N1,N5, and N10-(*Z*)-tri-p-coumaroylspermidine, (**2**): N1,N5-(*Z*)-N10-(*E*)-tri-p-coumaroylspermidine, (**3**): N1-(*E*)-N5-(*Z*)-N10-(*E*)-tri-p-coumaroylspermidine, and (**4**): N1,N5,N10-(*E*)-tri-p-coumaroylspermidine, were measured and counted with the previous method. The total content of the four compounds in the CSE and the yield are shown in **Table 1**. **Figure 3** showed the HPLC chromatogram of CSE. In every preparation, the total CSE coumaroylspermidine content was ~65% and the yield was up to 85%.

**Table 1:** Data for the preparation of CSE from Safflower Injection residues.

No.	Weight of residues (g)	Weight of CSE (g)	Total coumaroylspermidine content of CSE (%)	Yield (%)
1	200	3.45	65.01	86.16
2	200	3.25	65.27	85.02
3	200	3.70	64.31	85.23
average	200	3.47 ± 0.18	64.86 ± 0.41	85.47 ± 0.50

### CSE Effect in CUMS Rats

**Figure 4:**
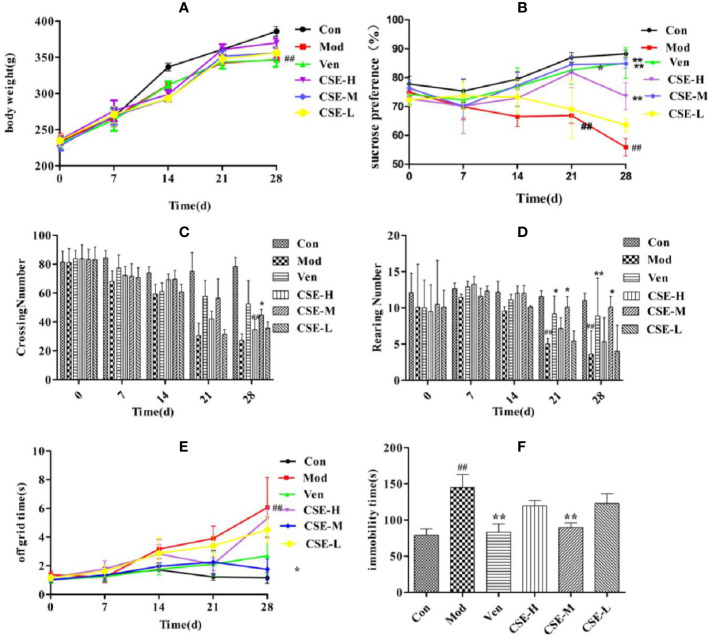
The effects of coumaroylspermidines extract (CSE) on body weight of CUMS rats **(A)**, sucrose preference **(B)**, crossing number **(C)**, rearing number **(D)**, off grid time **(E)**, and FST **(F)**. Values given are the mean ± SEM (n = 10). ^##^P < 0.01 compared with the control group. **P < 0.01, *P < 0.05 compared with the model group. (Con: control group, Mod: model group, Ven: venlafaxine group, CSE-H: CSE high group, CSE-M: CSE middle group, CSE-L: CSE low group).

The effects of CSE on the body weight of CUMS rats are shown in **Figure 4A**. Rats in each group showed no significant difference in body weight after 1 week of adaptation. After four weeks of CUMS procedures, rats in the Mod group showed lower body weight than the Con group (*P* < 0.01). Rats in the CSE-H and CSE-M groups showed a trend of increasing body weight compared to the Mod group, but no significance. It prompted that CSE may influence the other factors rather than food intake.

The SPT was used to exam the CSE effects on rats who underwent CUMS procedures (**Figure 4B**). Rats subjected to the 28-day stress procedures in the Mod group displayed lower sucrose preference than the Con non-stressed rats, confirming successful implementation of the CUMS model (*P* < 0.01). The Ven group (positive control) displayed significantly higher sucrose preference in CUMS rats compared to the Mod group (85.07%, *P* < 0.01). Similarly, prophylactic oral administration of CSE resulted in restoration of sucrose preference to normal levels in CUMS rats in the CSE-M group (84.86%, *P* < 0.01) and the CSE-H group (73.61%, *P* < 0.01). The restoration of sucrose preference effects for CSE-M and CSE-H were 99.75% and 86.53% of the Ven group, respectively. The CSE-L group trended towards reversion to sucrose preference in CUMS rats, though this was not significant compared to the Mod group (63.62%, *P* = 0.135).

The effects of CSE on the number of crossing and rearing in CUMS rats was investigated using the OFT (**Figures 4C, D**). After 4 weeks of exposure to CUMS, there were fewer crossings and rearings by rats in the Mod group than the Con group. Ven treatment increased the number of crossings and rearings compared to the Mod group (*P* < 0.01). Daily oral administration of CSE-M showed a significant effect on the increasing number of crossings and rearings in comparison to the Mod group (*P* < 0.05). The increasing effect for CSE-M was up to 85.60% and 86.37% of Ven group. The time out of the central grid was as one of the indexes from OFT, which indicates the animals' curiosity and adaptability to the novel environment with similarity to depression patients' fear of strange environments. Compared with the Con group, the Mod group spent more time off the grid after 28-day of CUMS procedures (**Figure 4E**). The time of leaving central grid was significantly lower in the Ven group. Chronic oral administration of CSE-M significantly decreased time spent in the central grid in CUMS rats. In other words, the middle dose of CSE can improve the adaptability of depression-like rats to strange environment. While the CSE-H and CSE-L groups trended towards less time spent in the central grid, which was relative to the Ven group with no statistical difference.

The FST was used to investigate the effects of CSE on immobility time in CUMS rats (**Figure 4F**). Following a 4-week CUMS regimen, immobility time in the Mod group was more extended than the Con group. Prophylactic administration of CSE was found to reduce immobility time in CUMS rats compared with the Mod group (*P* < 0.01). The CSE-M group showed the most obvious effect, and immobility time was lower than the Mod group (*P* < 0.01), which reached 93.03% of Ven group.

### CSE Regulates the Levels of HPA Axis-Related Hormones in CUMS Rats

**Figure 5:**
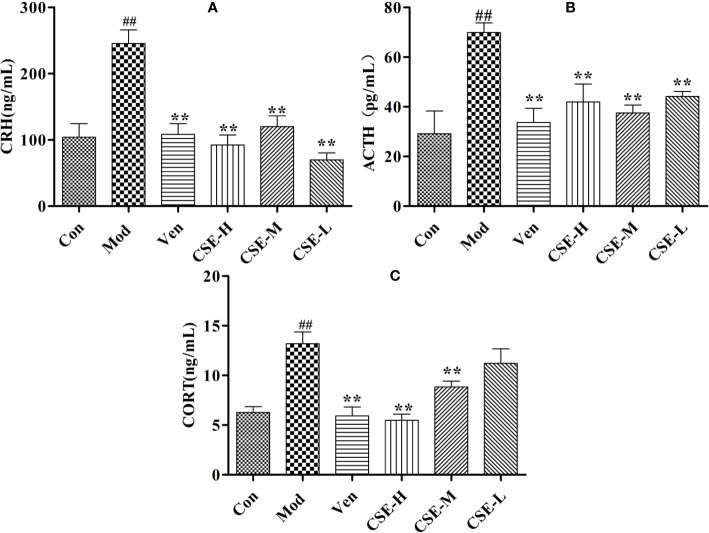
The effects of CSE on HPA axis of CUMS rats. **(A)** Corticotropin-Releasing Hormone (CRH), **(B)** Adrenocorticotropic Hormone (ACTH), **(C)** Corticosterone (CORT). Values given are the mean ± SEM (n = 10). ^##^*P* < 0.01 compared with the control group. **P < 0.01 compared with the model group.

The levels of hormones CRH, ACTH, and CORT in the rat serum related to the HPA axis were evaluated by ELISA. As shown in **Figure 5**, the serum levels of CRH, ACTH, and CORT in the Mod group were significantly higher than those in the Con group (*P* < 0.01). The expression levels of CRH were markedly lower the drug-treated groups compared with the Mod group (*P* < 0.01). After oral administration of CSE, all groups sh1owed a reduction in CRH levels significantly compared to the Mod group (*P* < 0.01). ACTH expression was significantly reduced in CUMS rats compared with those in the Mod group. All dose groups of CSE can improve the reduced ACTH with significance. Compared to the Con group, rats treated with chronic 28-day CUMS treatment showed significantly increased CORT levels in CUMS rats, and those treated with anti-depressants in the Ven group displayed significant alterations in increasing of CORT levels. To sum up, CSE can ameliorate the HPA axis disorder of depression-like rats.

### Effect of CSE on Neurotransmitters in Hippocampus of CUMS Rats

**Figure 6:**
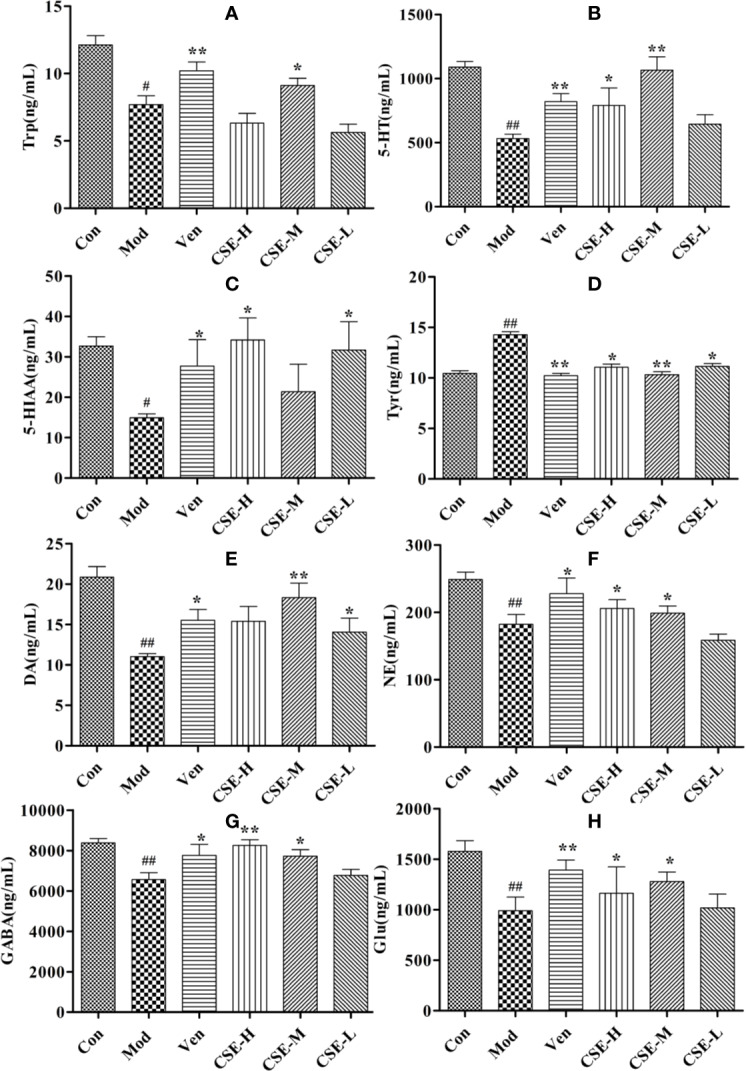
The concentrations of CSE on monoamine neurotransmitter in hippocampus of rats, **(A)** Trp, **(B)** 5-HT, **(C)** 5-HIAA, **(D)** Tyr, **(E)** DA, **(F)** NE, **(G)** GABA, and **(H)** Glu. Values given are the mean ± SEM (n = 10). ^##^*P* < 0.01, ^#^*P* < 0.05 compared with the control group. **P < 0.01, *P < 0.05 compared with the model group.

The effects of CSE on levels of the monoamine neurotransmitters Trp, 5-HT, and 5-HIAA in the hippocampus of CUMS rats were investigated and showed in **Figures 6A–C**. Hippocampal Trp levels were significantly lower in the Mod group than the Con group. The Ven group (positive control) showed significantly higher Trp levels than the Mod group. Oral administration of CSE-M for 28 days significantly altered the decreasing content of Trp in the hippocampus compared to the Mod group (*P* < 0.01). Hippocampal levels of 5-HT were significantly lower in Mod group than the Con group after 4 weeks of CUMS procedures. A significant increase in hippocampal 5-HT levels was observed after chronic oral administration of CSE-H and CSE-M compared to the Mod group (*P* < 0.01). After 4 weeks of oral administration of CSE-H and CSE-L, hippocampal 5-HIAA levels were increased in CUMS rats compared to the Mod group (*P* < 0.05).

The effects of CSE on levels of neurotransmitters in the NE metabolic pathway in the hippocampus of CUMS rats were also investigated and showed in **Figures 6D–F**. Levels of Try were significantly lower in the Mod group than in the Con group and showed a significant increase after 28 days of CSE-H or CSE-M administration (*P* < 0.05). Levels of DA in the Mod group, which were subjected to 4 weeks of CUMS procedures were significantly lower than in the Con group. Compared to the Mod group, daily oral administration of CSE-M and CSE-H significantly altered DA concentrations in CUMS rats (*P* < 0.05). Long-term administration of CSE resulted in a significant increase of NE concentration in CUMS rats compared with the Mod group.

The levels of GABA and Glu were significantly decreased in CUMS rats compared to those in the Con group (**Figures 6G, H**). After chronic oral administration of CSE-M and CSE-H, levels of GABA and Glu were seen to higher than the Mod group (*P* < 0.05).

### CSE Altered Metabolic Profiles in CUMS Rats

#### Identification of Serum Aqueous Metabolites

**Figure 7:**
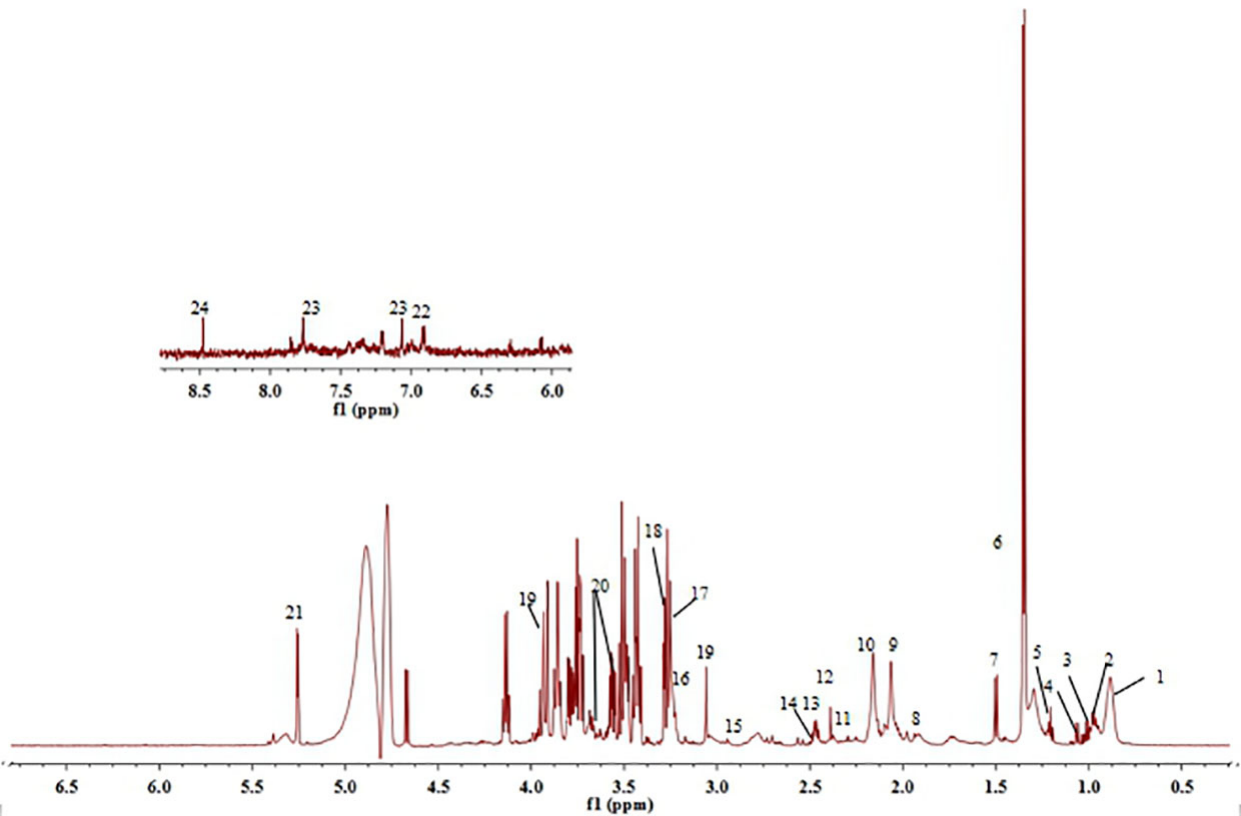
^1^H-NMR spectra of serum obtained from control group. (1) Lipid, (2) Leucine, (3) Valine, (4) Isoleucine, (5) β-hydroxybuty acid, (6) Lactic acid, (7) Alanine, (8) Acetate, (9) N-acetylglycoprotein, (10) O-acetylglycoprotein, (11) Acetoacetate, (12) Glutamic acid, (13) Pyruvate, (14) Glutamine, (15) Dimethylamine, (16) Choline, (17) Phosphatidylcholine, (18) Trimethylamine oxide, (19) Creatine, (20) Glycerin, (21) α-Glucose, (22) Tyrosine, (23) Histidine, and (24) Formate.

The serum of each group of rats was collected and tested for ^1^H-NMR spectrum. **Figure 7** is a typical ^1^H-NMR spectrum of the serum metabolites of the control group. By comparison with the database, a total of 24 metabolites, including amino acids, lipids, choline, and carbohydrates, were identified in the serum of the control group. Detail information of metabolites were shown in **Table 2**.

**Table 2:** ^1^H-NMR assignments of major metabolites in serum of rats.

No.	Metabolites	Moieties	Chemical shift (δ_H_)
1	Lipid	CH_3_, (CH_2_)_n_	0.87 (m),1.29 (m)
2	Leucine	α CH, β CH_2_, γ CH_3_, δ CH_3_	0.96 (d, *J*=7.1 Hz), 0.97 (d, *J*=6.6 Hz), 3.65 (d),1.95 (m),0.94 (t), 1.02 (s)
3	Valine	γ CH_3_, γ CH_3_	0.99 (d, *J*=6.6 Hz), 1.04 (d, *J*=7.2 Hz)
4	Isoleucine	δ CH_3_, δ CH_3_, δ CH_3_, γ CH_3_, γ CH_2_	0.94 (t, *J*=7.4 Hz), 1.01 (d, *J*=7.0 Hz), 1.27 (m)
5	β-Hydroxybuty acid	γ CH_3_	1.20 (d, *J*=6.6 Hz), 2.41 (d, *J*=6.6 Hz)
6	Lactic acid	α CH, β CH_3_	1.33 (d, *J*=8.4 Hz), 4.12 (d, *J*=8.3 Hz)
7	Alanine	β CH_3_	1.48 (d, *J*=8.6 Hz)
8	Acetate	CH_3_	1.92 (s)
9	N-acetylglycoprotein	CH_3_	2.04 (s)
10	O-acetylglycoprotein	CH_3_	2.14 (s)
11	Acetoacetate	CH_3_	1.92 (s)
12	Glutamic acid	α CH, β CH_2_	2.35 (m)
13	Pyruvate	CH_3_	2.37 (s)
14	Glutamine	α CH, β CH_2_	2.14 (m), 2.46 (m)
15	Dimethylamine	N CH_3_	2.93 (s)
16	Choline	N (CH_3_)_3_	3.20 (s)
17	Phosphatidylcholine	N (CH_3_)_3_	3.21 (s)
18	Trimethylamine oxide	CH_3_	3.26 (s)
19	Creatine	CH_3,_ CH_2_	3.04 (s), 3.94 (s)
20	Glycerin	CH_2_OH, CH_2_OH	3.66 (dd, *J*=11.4, 4.2 Hz), 3.56 (dd, *J*=9.6, 3.6 Hz)
21	α-Glucose	1-CH	5.23 (d, *J*=4.2 Hz)
22	Tyrosine	3 or 5-CH, 2 or 6-CH	6.89 (d), 7.18 (m)
23	Histidine	2-CH, 4-CH	7.74 (s), 7.04 (s)
24	Formate	CH	8.45 (s)

(s, singlet; d, doublet; t, triplet; q, quarter; m, multiplet; dd, doublet of doubles).

#### Effect of CSE on Serum of CUMS Rats and Analysis of Potential Metabolites

**Figure 8:**
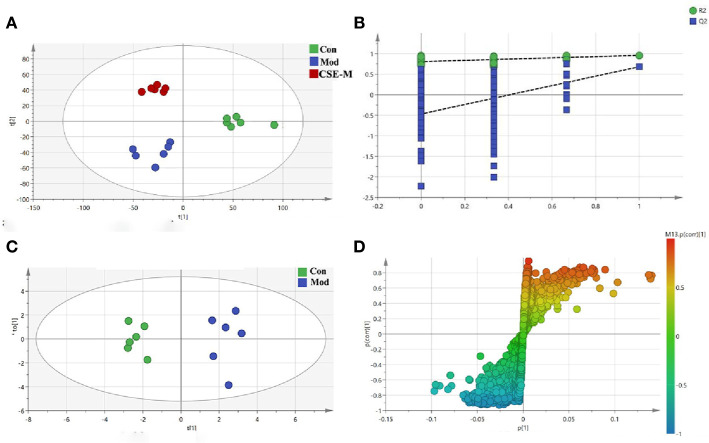
Multivariate analyses of serum 1H-NMR spectra data. **(A)** PLS-DA plots derived from 1H-NMR spectra on serum of rats in control, model and CSE-M groups. (control group 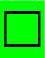, model group 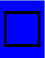, CSE-M group 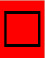), **(B)** PLS-DA model validation diagram, **(C)** OPLS-DA scatters plot on serum of rats in control and model groups (Con group 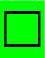, Mod group 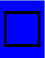), **(D)** corresponding S-plot of OPLS-DA.

In order to obtain the potential metabolites, a supervised PLS-DA method was first used to analyze the serum ^1^H-NMR data of the Con group, Mod group and CSE-M group (**Figure 8A**). Each dot represented a separate sample, and the clusters corresponding to the different groups' metabolic patterns were shown in the score plot. In **Figure 8A**, the model group and the control group are clearly separated, indicating that the CUMS model is successfully constructed, and the difference in metabolites between this two groups is significant. The CSE-M group was situated between the Mod group and the Con group in **Figure 8A**, further indicating that CSE could regulate metabolic disorder caused by CUMS. R2 and Q2 permutation test showed the validation of PLS-DA model (**Figure 8B**). R2Y was 0.983, R2X was 0.983, and Q2 was 0.562. These data showed that the model is well and there is no overfitting.

**Figure 9:**
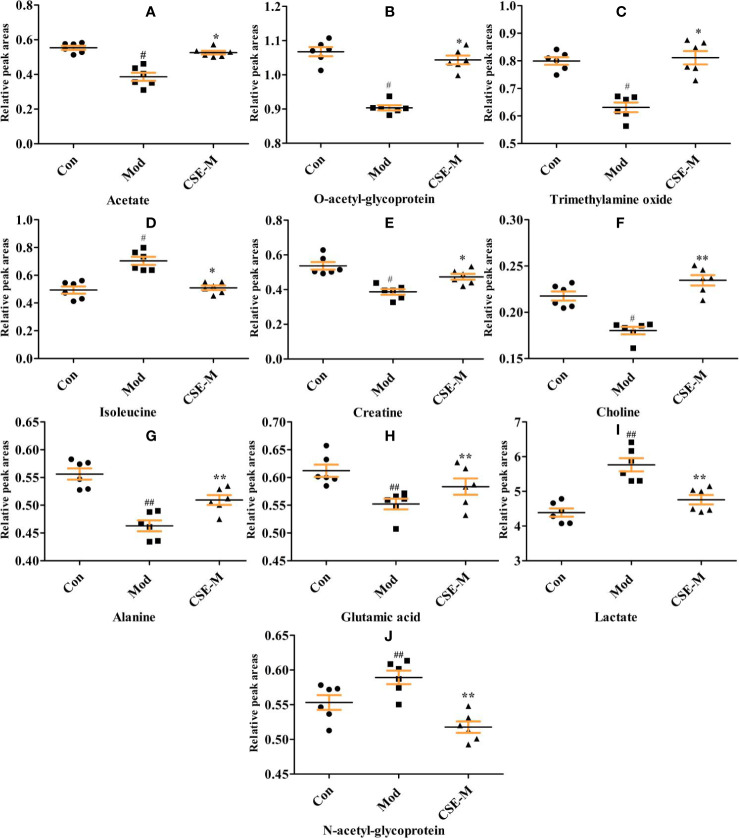
Comparison of relative peak areas of the potential metabolites in 1H-NMR associated with CSE treatment in serum. The data are presented as means ± SEM (n = 6). ^##^P < 0.01, ^#^P < 0.05 compared with the control group. **P < 0.01, *P < 0.05 compared with the model group. The 10 metabolites were: **(A-J)** acetate, O-acetyl-glycoprotein, trimethylamine oxide, isoleucine, creatine, choline, alaline, glutamic acid, lactate, N-acetyl-glycoprotein.

Next, an OPLS-DA method was used to identify the differential metabolites between the Mod group and the Con group. As shown in **Figure 8C**, the model group is clearly separated from the control group in the OPLS-DA score plots, further indicating that the model was successfully established. Meanwhile, the VIP values of S-plots (>1.0) and T-tests (p < 0.05) were used to screen the significantly differential metabolites. As shown in **Figure 9**, seven potential metabolites (acetate, O-acetyl-glycoprotein, trimethylamine, creatine, choline, alanine, and glutamic acid) in Mod rats were significantly reduced compared to the Con group, and three potential metabolites (isoleucine, lactate, and N-acetyl-glycoprotein) were increased. Importantly, CSE-M can regulate these 10 potential metabolites close to the control level (**Figure 9**), which shows that CSE has a good antidepressant effect.

#### Metabolic Pathway Analysis

**Figure 10:**
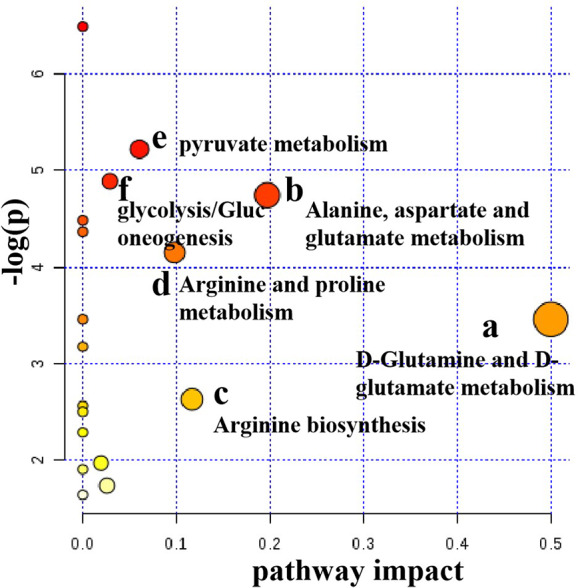
Pathway analysis of serum metabolites affected by CSE.

Metabolic pathway analysis can not only obtain information on the biology of metabolites in disease, but also provide important information for the pathophysiology of disease. The possible metabolic pathways of depression were obtained after the ten potential endogenous biomarkers importing to into the MetaboAnalyst. Pathway analysis of metabolites affected by CSE was presented in **Figure 10**. With impact value > 0.1, those pathways were considered to be the most relevant ones associated with CUMS-induced depression-like behavior in rats. Four pathways, (1) D-glutamine and D-glutamate metabolism, (2) Alanine, aspartate and glutamate metabolism, (3) Arginine biosynthesis, and (4) Arginine and proline metabolism were identified to be the most effected metabolic pathways associated with CUMS (**Figure 10**). This pathway analysis indicates that the antidepressant effect of CSE on CUMS rats may be related to these four pathways.

### Correlation Between Monoamine Neurotransmitters and HPA Axis Hormones and Differential Metabolites

**Figure 11:**
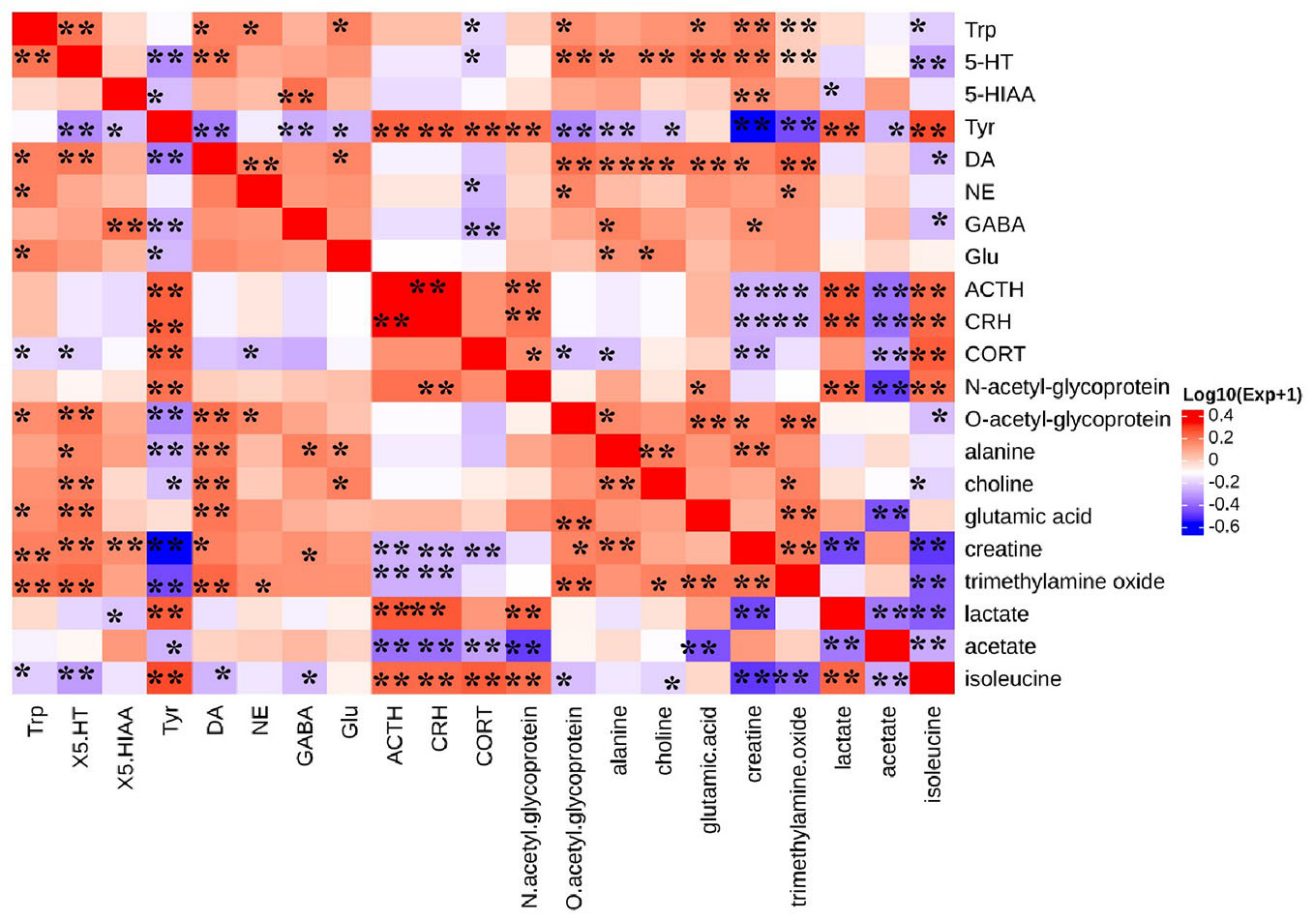
Heat map summarizing the Pearson's correlation between monoamine neurotransmitter, HPA axis hormones, and differential metabolites. (*P < 0.05, **P < 0.01).

We investigate the functional relationship between altered monoamine neurotransmitters, HPA axis hormones and differential metabolites using Pearson's correlation coefficients (Li et al., 2018). The coefficients demonstrated that 5-HT was negatively correlated with isoleucine, while the HPA-axis hormones were positively correlated with isoleucine (**Figure 11**). In addition, glutamic acid was positively correlated with Trp, 5-HT, and DA. Trimethylamine oxide, ACTH, and CRH showed a negative correlation, while 5-HT, Trp, and DA showed a positive correlation. Levels of branched-chain amino acids (BCAAs), such as isoleucine were significantly reduced in the serum of CUMS rats, indicating impeded 5-HT release in the brain (Blomstrand, 2006; Noh et al., 2006). Additionally, we found that levels of isoleucine and 5-HT returned to normal following CSE treatment.

## Discussion

In this study, we found that the sucrose preference of CUMS rats in the CSE-M and CSE-H groups could considerably increase (*P* < 0.01), but only CSE-M showed a significant effect on the open field experiment (*P* < 0.05) and forced swimming tests (*P* < 0.01) of CUMS rats. This result indicated that diverse components with different contents in CSE have different influence on the depression-like behavior of CUMS rats. Thus, qualitative and quantitative chemical analysis on CSE and more experimental verification of the antidepressant effect need to be further performed.

Increased HPA activity is one of the common neurobiological abnormalities in patients with depression. CRH is released from the paraventricular nucleus of the hypothalamus and enhances the release of pituitary ACTH during stress. Subsequently, this hormone enhances glucocorticoid secretion from the adrenal cortex. According to this interpretation, it was observed that stressful rats had higher levels of CRH, ACTH, and CORT. However, CSE and venlafaxine treatment could reverse these indices, indicating that the neuroendocrine system had improved. In addition, hippocampal monoaminergic neurons may be damaged due to overactivity of the HPA axis, resulting in a reduction in monoamines (Zhang et al., 2019). After taking CSE and venlafaxine, the levels of 5-HT, DA, GABA, and NE were restored. Monoamines are key neuromodulators in the development of mood disorders. Studies have found that monoamine levels in brain regions (e.g., prefrontal cortex and hippocampus) can be increased by supplementing with antidepressants (Blier, 2016). This shows that the antidepressant effect of CSE may be related to the regulation of the monoaminergic system.

Glutamate is one of the main endogenous excitatory neurotransmitters, which plays an important role in the central nervous system. Glutamate cycle disorder is closely related to the occurrence and development of depression. In this study, compared with the Con group, the glutamine level of Mod group was significantly reduced. The reason may be related to the inhibition of the TCA cycle, or to the reversal of glutamate transporters and the affected glutamate-glutamine cycle (Liu et al., 2019). However, this decreased trend was restored after taking CSE. It is suggested that the metabolic pathways of glutamine and D-glutamate may play an important role in the antidepressant effect of CSE.

Although the structure of the main components has three more coumaroyl groups in CSE than spermidine, these three substituents are easily degraded to spermidine *in vivo*. Spermidine is a rich natural polyamine present in all organisms, including many herbs used in TCM (*Lycium barbarum*, *Astragalus propinquus*, etc). Spermidine is often taken in our daily diet, and supplementation of spermidine in the diet can extend life span and health span (Eisenberg et al., 2016; Yue et al., 2017; Madeo et al., 2018). In animal models, it has significant cardioprotective and neuroprotective effects, improves metabolic decline associated with aging and stimulates anticancer immunosurveillance. Autophagy plays an essential role in the health function of spermidine (Madeo et al., 2018). After taking spermidine, the aPWV (aortic pulse wave velocity) of the aged mice became normal, and NO-mediated endothelial-dependent relaxation was restored. This effect of spermidine is thought to be related to autophagy (LaRocca et al., 2013). Spermidine also prevents the toxicity of transgenic α-synuclein expression in flies and nematodes (Buettner et al., 2014), which is consistent with the idea that (spermidine-enhanced) autophagy clear aggregates of spolyubiquitin-associated and other neurotoxic protein. Autophagy is a lysosomal-dependent protein degradation pathway that maintains the homeostasis of the cellular environment through the degradation and recycling of damaged organelles and longevity proteins. It works with the ubiquitin proteasome system to maintain normal metabolism. There is still much controversy about autophagy showing a positive or negative regulatory effect on neurological diseases (Son et al., 2012), but some animal experimental results suggest that the anti-depression effect of some antidepressants may be related to its up-regulation of cellular autophagy. Antidepressants amitriptyline and citalopram increase rat astrocyte and neuron autophagy levels (Zschocke et al., 2011). Ketamine did not show the anti-depression effect on FKBP51 knockout mice, but the expression of autophagy markers (such as Beclin-1) in wild-type mice was upregulated after ketamine treatment, while Beclin-1 was not upregulated in FKBP51 knockout mice (Gassen et al., 2014). In addition, studies suggesting that autophagy promotes synapse development in Drosophila (Shen and Ganetzky, 2009).

Spermidine also inhibits pro-inflammatory cytokines and improves the bioavailability of arginine, which is needed for nitric oxide (NO) bio-synthesis. In the LPS-stimulated RAW 264.7 macrophages, spermidine significantly attenuated the LPS-induced production of NO and PGE2 by downregulating iNOS and COX-2 expression on both the protein and mRNA levels without cytotoxicity (Jeong et al., 2018). In the rotenone induced Parkinson's disease rat model, not only the levels of IL-6, IL-1β, and TNF-α in striatum were improved, but the level of GABA, DA, NE, and 5-HT was significantly increasing when treatment with spermidine compared with model group (Sharma et al., 2018). Findings from our study also suggests that 28-day administration of CSE can restore the downward trend of these neurotransmitters caused by CUMS. Increasing evidence suggests that inflammatory reactions are involved in the pathophysiology of depression (Hashimoto, 2015; Mechawar and Savitz, 2016; Miller et al., 2017). A meta-analysis showed that the levels of pro-inflammatory cytokines interferon-γ, IL-2, IL-6, and TNF-α in the blood of untreated or drug-treated depression patients are increased compared to healthy controls (Köhler et al., 2018). Many selective serotonin reuptake inhibitors (SSRIs), such as escitalopram, can significantly block LPS-induced increase in serum pro-inflammatory cytokine TNF-α levels and increase IL-10 levels to exert antidepressant effects (Dong et al., 2016).

Nevertheless, spermidine is an abundant natural polyamine present in our food that can prolongs health span and life span by multiple ways. And this is first time to report an herb extract (CSE) from safflower residues displaying an anti-depressant effect in CUMS model. Autophagy and anti-inflammatory may be the main mechanisms for CSE.

## Conclusion

Safflower injection is approved for the treatment of cardiovascular diseases in China. However, a large amount of drug residues is yielded during the production process due to a water extraction and alcohol precipitation process. In this study, a total coumaroylspermidine extract (CSE) including four coumaroylspermidine compounds was prepared from safflower injection residues for the first time. The total coumaroylspermidine content of CSE was determined to be 64.86 ± 0.41%. CSE exhibits a significant anti-depressant effect in CUMS model, which are probably related to regulation of the HPA axis, monoamine neurotransmitters, and D-glutamine and D-glutamate metabolism. This study not only prepared an herb residue extract with antidepressant effects but also provided a way for the efficient utilize of safflower residues. And the clear mechanism of CSE antidepressant effect and possible side effects remains to be investigated in a suitable experimental setting.

## Data Availability Statement

All datasets generated for this study are included in the article/**Supplementary Material**.

## Author Contributions

JT and TL designed and completed the experiments. TL accomplished all experiments, including wrote the paper and analyzed data. YJ achieved the separation of CSE. SL was responsible for the design of the entire experiment and wrote the paper. LZ and XQ grasp the overall idea and gave the technical guidance. All authors participate in the results discussion and article modification.

## Funding

This paper was supported by the Key Research and Development Project of Shanxi Province (201603D3113008, 2016ZD0504, 201903D321210 and 201803D31031). Project of Standard for TCM (ZYBZH-C-JIN-44), and the National Natural Science Fund of China (31800293). We thank the service provided by the Instrument Center of Shanxi University.

## Conflict of Interest

The authors declare that the research was conducted in the absence of any commercial or financial relationships that could be construed as a potential conflict of interest.

## References

[B1] Ao H. Feng W. Peng C. (2018). Hydroxysafflor yellow A: a promising therapeutic agent for a broad spectrum of diseases. Evid-Based. Compl. Alt. 2018, 1–17. 10.1155/2018/8259280. article ID: 8259280. PMC6176289 30356354

[B2] Blier P. (2016). Neurobiology of depression and mechanism of action of depression treatments. J. Clin. Psychiatry 77, e319. 10.4088/JCP.13097tx3c 27046319

[B3] Blomstrand E. (2006). A role for branched-chain amino acids in reducing central fatigue. J. Nutr. 136, 544S–547S. 10.1093/jn/136.2.544S 16424144

[B4] Buettner S. Broeskamp F. Sommer C. Markaki M. Habernig L. Alavian-Ghavanini A. (2014). Spermidine protects against α- synuclein neurotoxicity. Cell Cycle 13, 3903–3908. 10.4161/15384101.2014.973309 25483063 PMC4614020

[B5] Dong C. Zhang J. C. Yao W. Ren Q. Yang C. Ma M. (2016). Effects of escitalopram, R-citalopram, and reboxetine on serum levels of tumor necrosis factor-alpha, interleukin-10, and depression-like behavior in mice after lipopolysaccharide administration. Pharmacol. Biochem. Behav. 144, 7–12. 10.1093/ijnp/pyw089 26892759

[B6] Eisenberg T. Knauer H. Schauer A. Büttner S. Ruckenstuhl C. CarmonaGutierrez D. (2009). Induction of autophagy by spermidine promotes longevity. Nat. Cell Biol. 11, 1305–1314. 10.1038/ncb1975 19801973

[B7] Eisenberg T. Abdellatif M. Schroeder S. Primessnig U. Stekovic S. Pendl T. (2016). Cardioprotection and lifespan extension by the natural polyamine spermidine. Nat. Med. 22, 1428–1438. 10.1038/nm.4222 27841876 PMC5806691

[B8] Gassen N. C. Zschocke J. Hafner K. Zellner A. Kollmannsberger L. Uhr M. (2014). Association of FKBP51 with priming of autophagy pathways and mediation of antidepressant treatment response: evidence in cells, mice, and humans. PloS Med. 11, e1001755. 10.1371/journal.pmed.1001755 25386878 PMC4227651

[B9] Gong W. Zhu S. Chen C. Yin Q. Li X. Du G. (2019). The anti-depression effect of *Angelicae Sinensis* radix is related to the pharmacological activity of modulating the hematological anomalies. Front. Pharmacol. 10, 192. 10.3389/fphar.2019.00192 30894817 PMC6414447

[B10] Hashimoto K. (2015). Inflammatory biomarkers as differential predictors of antidepressant response. Int. J. Mol. Sci. 16, 7796–7801. 10.3390/ijms 16047796 25856677 10.3390/ijms16047796 PMC4425050

[B11] Jeong J. W. Cha H. J. Han M. H. Hwang S. J. Lee D. S. Yoo J. S. (2018). Spermidine protects against oxidative stress in inflammation models using macrophages and zebrafish. Biomol. Ther. 26, 146–156. 10.4062/biomolther.2016.272 PMC5839493 28365977

[B12] Jin Y. Li S. Zhang L. (2018). Study on extraction process and content determination of spermidines in Safflower Injection dregs. Chem. Res. Appl. 30, 1239–1245. 10.3969/j.issn.1004-1656.2018.08.006

[B13] Joca S. R. Moreiane F. A. Wegener G. (2015). Atypical neurotransmitters and the neurobiology of depression. CNS Neurol. Disord-DR. 14, 1001–1011. 10.2174/1871527314666150909114804 26350337

[B14] Köhler C. A. Freitas T. H. Stubbs B. Maes M. Solmi M. Veronese N. (2018). Peripheral alterations in cytokine and chemokine levels after antidepressant drug treatment for major depressive disorder: systematic review and meta-analysis. Mol. Neurobiol. 55, 4195–4206. 10.1007/s12035-017-0632-1 28612257

[B15] LaRocca T. J. Gioscia-Ryan R. A. Hearon C. M. J. Seals D. R. (2013). The autophagy enhancer spermidine reverses arterial aging. Mech. Ageing Dev. 134, 314–320. 10.1016/j.mad.2013.04.004 23612189 PMC3700669

[B16] Li S. Yuan M. Zhang L. (2016). Simultaneous determination of four coumaroylspermidine constituents in *Carthamus tinctorius* by HPLC-DAD. Zhongguo Zhong Yao Za Zhi 41, 1480–1484. 10.4268/cjcmm20160819 28884543

[B17] Li Y. Peng Y. Ma P. Yang H. Xiong H. Wang M. (2018). Antidepressant-like effects of *Cistanche tubulosa* extract on chronic unpredictable stress rats through restoration of gut microbiota homeostasis. Front. Pharmacol. 9, 967. 10.3389/fphar.2018.00967 30186183 PMC6112285

[B18] Liu M. H. Wang Z. Y. Chen L. Liu G. C. Zheng H. (2016). Application of peanut shell and Chinese medicine mixed biochar as soil amendment to lead contaminated soil. Period. Ocean Univ. China. 46, 101–107. 10.16441/j.cnki.hdxb.20150035

[B19] Liu X Zheng X Du G Li Z Qin X (2019). Brain metabonomics study of the antidepressant-like effect of Xiaoyaosan on the CUMS-depression rats by 1H NMR analysis. J. Ethnopharmacol. 235, 141–154. 10.1016/j.jep.2019.01.018 30708033

[B20] Ma J. Chen Y. Zhao Y. Chen D. Wang H. (2019). Effects of traditional Chinese medicine residue on plant growth and soil properties: a case study with maize (*Zea mays L*.). Environ. Sci. Pollut. R. 26, 32880–32890. 10.1016/j.still.2019.104386 31502056

[B21] Madeo F. Eisenberg T. Pietrocola F. Kroemer G. (2018). Spermidine in health and disease. Science 359, 410. 10.1126/science.aan2788 29371440

[B22] Mechawar N. Savitz J. (2016). Neuropathology of mood disorders: do we see the stigmata of inflammation. Transl. Psychiatry 6, e946. 10.1038/tp.2016.212 27824355 PMC5314124

[B23] Miller A. H. Haroon E. Felger J. C. (2017). Therapeutic implications of brain-immune interactions: treatment in translation. Neuropsychopharmacol. 42, 334–359. 10.1038/npp.2016.167 PMC5143492 27555382

[B24] Noh H. S. Hah Y. S. Nilufar R. Han J. Bong J. H. Kang S. S. (2006). Acetoacetate protects neuronal cells from oxidative glutamate toxicity. J. Neurosci. Res. 83, 702–709. 10.1002/jnr.20736 16435389

[B25] Palazidou E. (2012). The neurobiology of depression. Brit. Med. Bull. 101, 127–145. 10.1093/bmb/lds004 22334281

[B26] Sharma S. Kumar P. Deshmukh R. (2018). Neuroprotective potential of spermidine against rotenone induced Parkinson's disease in rats. Neurochem. Int. 116, 104–111. 10.1016/j.neuint.2018.02.010 29501454

[B27] Shen W. Ganetzky B. (2009). Autophagy promotes synapse development in Drosophila. J. Cell Biol. 187, 71–79. 10.1083/jcb.200907109 19786572 PMC2762098

[B28] Son J. H. Shim J. H. Kim K. H. Ha J. Y. Han J. Y. (2012). Neuronal autophagy and neurodegenerative diseases. Exp. Mol. Med. 44, 89–98. 10.3858/emm.2012.44.2.031 22257884 PMC3296817

[B29] Tian J. Shi B. Xiang H. Gao S. Qin X. (2013). Du, G.H. ^1^H-NMR-based metabonomic studies on the anti-depressant effect of Genipin in the chronic unpredictable mild stress rat model. PloS One 8, e75721. 10.1371/journal.pone.0075721 24058700 PMC3776757

[B30] Tian J. Liu C. Xiang H. Zheng X. Peng G. Zhang X. (2015). Investigation on the antidepressant effect of sea buckthorn seed oil through the GC-MS-based metabolomics approach coupled with multivariate analysis. Food Funct. 6, 3585. 10.1039/c5fo00695c 26328874

[B31] Tian X. Yang T. He J. Chu Q. Jia X. Huang J. (2017). Fungal community and cellulose-degrading genes in the composting process of Chinese medicinal herbal residues. Bioresour. Technol. 241, 374–383. 10.1016/j.biortech.2017.05.116 28578278

[B32] Wang H. Feng F. (2009). Identification of components in Zhi-Zi-Da-Huang decoction by HPLC coupled with electrospray ionization tandem mass spectrometry, photodiode array and fluorescence detectors. J. Pharmaceut. Biomed. Anal. 49, 1157–1165. 10.1016/j.jpba.2009.02.023 19356878

[B33] Wang K. Li S. Zhao Y. Li H. Zhang L. W. (2018). In vitro anticoagulant activity and active components of Safflower Injection. Molecules 23, 170. 10.3390/molecules23010170 PMC6017571 29342933

[B34] Youn H. Noh J. (2001). Screening of the anticoccidial effects of herb extracts against *Eimeria tenella*. Vet. Parasitol. 96, 257–263. 10.1016/s0304-4017(01)00385-5 11267752

[B35] Yuan M. Li S. Zhang L. (2015). Isolation and purification of coumaroylspermidines from *Carthamus tinctorius L*. and their inhibition effects on [^3^H]-5-HT reuptake. J. Shanxi Med. Univ. 46, 442–447. 10.13753/j.issn.1007-6611.2015.05.014

[B36] Yue F. Li W. Zou J. Jiang X. Xu G. Huang H. (2017). Spermidine prolongs lifespan and prevents liver fibrosis and hepatocellular carcinoma by activating MAP1S-mediated autophagy. Cancer Res. 77, 2938–2951. 10.1158/0008-5472 28386016 PMC5489339

[B37] Zhang L. Gu J. Wang X. Sun W. Yin Y. Sun Y. (2017). Behavior of antibiotic resistance genes during co-composting of swine manure with Chinese medicinal herbal residues. Bioresour. Technol. 244, 252–260. 10.1016/j.biortech.2017.07.035 28780258

[B38] Zhang L. L. Yang Z. Y. Fan G. Ren J. N. Yin K. J. Pan S. Y. (2019). Antidepressant-like effect of *Citrus sinensis* (L.) Osbeck essential oil and its main component limonene on mice. J. Agric. Food Chem. 67, 13817–13828. 10.1021/acs.jafc.9b00650 30905156

[B39] Zhou Y. Selvam A. Wong J. W. C. (2014). Evaluation of humic substances during co-composting of food waste, sawdust and Chinese medicinal herbal residues. Bioresour. Technol. 168, 229–234. 10.1016/j.biortech.2014.05.070 24951275

[B40] Zhou Y. Selvam A. Wong J. W. C. (2018). Chinese medicinal herbal residues as a bulking agent for food waste composting. Bioresour. Technol. 249, 182–188. 10.1016/j.biortech.2017.09.212 29040853

[B41] Zschocke J. Zimmermann N. Berning B. Ganal V. Holsboer F. Rein T. (2011). Antidepressant drugs diversely affect autophagy pathways in astrocytes and neurons-dissociation from cholesterol homeostasis. Neuropsychopharmacol. 36, 1754–1768. 10.1038/npp.2011.57 PMC3138654 21508931

