# Supplementary material for: Antidepressant-Like Effects of Coumaroylspermidine Extract From Safflower Injection Residues
Source: Front Pharmacol. 2020 Jun 17;11:713. doi: 10.3389/fphar.2020.00713 (PMC7311797; doi:10.3389/fphar.2020.00713)
Supplement: Supplementary file 1 [file DataSheet_1.docx]

**Supporting Materials**

**Antidepressant-Like Effects of the Coumaroylspermidine Extract from a Traditional Chinese Medicine (Safflower Injection) Residues**

Shi-Fei Li ^a,*^, Ting Li ^a^, Yu-Fang Jin ^a^, Xue-Mei Qin ^b^, Jun-Sheng Tian ^b,*^, Li-Wei Zhang ^a,*^

^a^Key Laboratory of Chemical Biology and Molecular Engineering of Education Ministry, Institute of Molecular Science, Shanxi University, Taiyuan 030006, PR China

^b^Modern Research Center for Traditional Chinese Medicine, Shanxi University, Taiyuan 030006, PR China

*Corresponding author. Tel.: +86-0351-7018113. Fax: +86-0351-7018113.

*email addresses*: [lisf@sxu.edu.cn](mailto:lisf@sxu.edu.cn);

1. Determination method of the total content of coumaroylspermidine compunds in CSE

This method was previously conducted by our group including sample preparation and method validation [1]. The dried powders CSE samples (1 mg) were accurately weighed and resolved in methanol to prepare the concentration to 0.05 mg/mL. Then the samples were filtered through 0.45 μm membrane before HPLC injection. Analyses were performed using an Agilent 1260 liquid chromatography system, equipped with a quaternary solvent delivery system, an autosampler and a DAD detector. Separation was carried out on a Zorbax XDB C18 column (250mm×4.6mm, 5 μm). The mobile phase consisted of solvent A (methanol) and solvent B (water) and the elution was 55% methanol. UV absorption was monitored at 270-300 nm. The column temperature was 25 ℃. The flow rate was 1.0 mL/min and sample injection volume was 10 μL. The calibration curves of the four coumaroylspermidine compounds is follows:

Table 1 Regression equation and linear range of coumaroylspermidines **1-4**

| coumaroylspermidine | regression equation | UV(nm) | R^2^ | linear range(mg/ml) |
| --- | --- | --- | --- | --- |
| **1** | Y=40317x-51.630 | 270 | 0.9991 | 0.0021-0.0416 |
| **2** | Y=35750x-29.313 | 280 | 0.9995 | 0.0026-0.0512 |
| **3** | Y=38854x-50.026 | 290 | 0.9996 | 0.0027-0.0540 |
| **4** | Y=48600x-67.042 | 300 | 0.9998 | 0.0050-0.1004 |

[1] Li, S.F.; Yuan, M.Y.; Zhang, L.W. Simultaneous determination of four coumaroylspermidine constituents in Carthamus tinctorius by HPLC-DAD. [Zhongguo Zhong Yao Za Zhi](https://www.ncbi.nlm.nih.gov/pubmed/?term=Simultaneous%20determination%20of%20four%20coumaroylspermidine%20constituents%20in%20Carthamus%20tinctorius%20by%20HPLC-DAD&utm_source=gquery&utm_medium=search) 2**016**, 41, 1480-1484.
